# Higher expression of inhibitory CD158b and CD158e NK cell receptor and age predicts treatment response in children with chronic hepatitis C

**DOI:** 10.1007/s00430-017-0526-x

**Published:** 2017-11-08

**Authors:** Anna Mania, Mariusz Kaczmarek, Paweł Kemnitz, Katarzyna Mazur-Melewska, Magdalena Figlerowicz, Jan Sikora, Wojciech Służewski, Jan Żeromski

**Affiliations:** 1 Department of Infectious Diseases and Child Neurology, University of Medical Sciences Poznan, Szpitalna 27/33, 60-572 Poznan, Poland; 2 Chair of Clinical Immunology, University of Medical Sciences Poznan, Poznan, Poland

**Keywords:** NK cell receptors, Antiviral treatment, Pegylated interferon-alpha, Chronic hepatitis C, Children

## Abstract

Treatment with pegylated interferon-α and ribavirin (PEG–IFN/RBV) is the only choice for chronic hepatitis C (CHC) in children. Natural killer (NK) cells were described to play a vital role in CHC. The aim of this study was to analyze the expression of peripheral blood NK cell receptors in their relation to PEG–IFN/RBV treatment response. Study included 26 children with CHC—13 boys, age range 13.42 ± 3.28 years. Blood for biochemical, virological and cytometric testing was taken for evaluation prior to the antiviral treatment. NK cell receptors were detected by flow cytometry and the results were presented as proportion of cells and mean fluorescence intensity (MFI). Therapy consisted of PEG–IFNα-2b (60 μg/m^2^ s.c 1×/week) and RBV (15 mg/kg p.o. daily). Treatment duration was response-related and varied from 12 to 72 weeks. Rapid virological response (RVR) was evaluated in the 4th week and sustained virological response (SVR) 6 months after completion of the therapy. RVR children were younger (11.67 ± 3.74 vs 15.35 ± 2.42; *p* = 0.001) and displayed higher CD158b (3.58 ± 0.16 vs 3.45 ± 0.13; *p* = 0.038) and CD158e expression (4.33 ± 0.21 vs 4.03 ± 0.16; *p* = 0.039). Density of CD158b (logMFI = 3.68 ± 0.22 vs 3.36 ± 0.16; *p* = 0.036) and CD158e expression was significantly higher (4.37 ± 0.14 vs 4.12 ± 0.21; *p* = 0.046) and NKG2D expression significantly lower (97.50 ± 3.46 vs 94.92 ± 5.93; *p* = 0.049) in SVR children. SVR children were also significantly younger (12.40 ± 3.66 vs 15.13 ± 2.83; *p* = 0.003). Significance of the age of patients, and expression of CD158b and CD158e were confirmed in univariate and multivariate analysis. Age of patients is negatively related to RVR and SVR. NK cell phenotype with higher expression density of CD158b and CD158e receptor was a positive predictor of SVR.

## Introduction

Hepatitis C virus (HCV) is affecting approximately 170 million people worldwide leading to serious consequences including chronic hepatitis (CHC), liver cirrhosis and hepatocelluar carcinoma [1]. HCV has developed numerous mechanisms to evade innate immune response in infected host leading to chronic infection and further complications. The immune system of the host was found to be responsible for the liver injury and the progression of the disease [2]. Natural killer (NK) cells are considered to be a major part of immune response against HCV infection. This type of cells does not require previous contact with the antigen to be able to recognize infected cells. NK cells produce interferon-ɤ, a cytokine of antiviral effect taking part in adaptive immune response. Moreover, NK cell possess cytotoxic capacity due to degranulation of cytotoxic granules containing perforin and granzyme. Finally, they may induce apoptosis of target cells up-regulating Fas ligand and tumor necrosis-related apoptosis inducing ligand on their surface [2]. Furthermore, it has been noticed that NK cell activity may be reduced in CHC patients [3]. Two major populations of NK cells were described: CD56+dim NK cells of mainly cytotoxic activity and CD56+bright cells mostly producing cytokines. NK cell regulation depends on the various number of inhibitory and activating receptors, that belong to natural cytotoxicity receptors or killer cell imunoglobulin-like receptors (KIR) [4]. KIRs are members of the CD158 gene family with activating and inhibitory potential, that modulate NK cell development and activity by interaction with class I major histocompatibility complex (MHC) [5].

KIRs consist of two (KIR2D) or three (KIR3D) extracellular immunoglobulin domains. Moreover, they have either short (S) or long (L) intracytoplasmatic tails responsible for the transduction of activating and inhibitory signals. Despite the fact that activating KIRs possess similar extracellular regions, their ligands are poorly defined in most instances. Each individual differs in the number and type of inherited KIRs. Interestingly, individual NK cells express one, few or none inhibitory KIRs. Furthermore, they may be coexpressed by subsets of NK cells. Nevertheless, inhibitory KIRs through interaction with autologous MHC class one prevent NK cells from killing normal cells [6, 7].

Growing evidence suggests that innate immunity influences the response to treatment in CHC [8, 9].

Until now, pegylated interferon-α and ribavirin (PEG–IFN/RBV) has been the only available therapy for CHC in children. In spite of existing new directly acting antivirals (DAA), interferon-based regimen is a part of daily clinical practice in a treatment of CHC in children. Thus significant proportion of patients does not respond to this type of therapy. Many prognostic factors were evaluated to search for baseline parameters enabling prediction of good response to PEG–IFN/RBV treatment [10, 11].

The aim of this study was to analyze baseline factors including the expression of peripheral blood NK cell activating and inhibitory receptors as predictors of the response to PEG–IFN/RBV treatment in children with CHC.

## Patients and methods

Study included 26 consecutive children with CHC—13 boys and 13 girls, mean age 13.42 ± 3.28 years. Children infected with HBV, HIV, HSV, CMV and EBV were excluded from the study as well as children with other chronic liver diseases. Informed consent for the participation in the study was obtained from the legal guardians of children. Duration of infection was counted from the moment of diagnosis. Children were enrolled to the study at least 5 years after completion of the oncological treatment if malignancy in the history was found. Period of 2 years was also obligatory in relation to previous antiviral treatment of CHC with recombinant IFN. Treatment experienced children were all nonresponders. Blood specimens for biochemical, virological testing were collected prior to the liver biopsy and in appropriate treatment intervals—in the 4th, 12th, 24th, 48th week of the therapy and 24th weeks of the follow-up period. Biochemical tests included alanine and asparatate aminotransferase activity (ALT and AST) and gammaglutamylotranspeptidase (GTP). Aminotransferase activity was evaluated using standard biochemical analyser. HCV-RNA was detected with RT-PCR test (Amplicor HCCTM test—sensitivity level 50 IU/ml). Genotype was evaluated with Versant HCV genotype test. General characteristic of the study and control group were presented in Table 1.

**Table 1:** Clinical and laboratory group characteristics at baseline

Parameter	Number (percent)	$\bar {X}$ ± SD	Control group	*p*
Age (years)	–	13.42 ± 3.28	13.48 ± 5.14	0.987
Gender: M/F	13/13 (50/50)	–	12/11 (52/48)	0.879
Age at diagnosis (years)	–	3.44 ± 4.23	–	–
Duration of infection (years)	–	10.58 ± 4.20	–	–
Probable route of infection: parenteral/vertical	22/4 (84.6/15.4)	–	–	–
History of malignancy Y/N	14/12 (53.8/46.15)		–	–
Former antiviral treatment Y/N	13/13 (50/50)	–	–	–
Weight (kg)	–	50.62 ± 15.38	61.83 ± 21.45	0.147
Body mass index—BMI (kg/m^2^)	–	19.68 ± 3.39	23.36 ± 6.11	0.015*
HCV genotype 1b/1a/3a	4/20/2 (15.4/76.9/7.7)	–	–	–
HCV-RNA (IU/ml)	–	1.75 × 10^5^±5.75 × 10^6^	–	–
ALT (IU/l)	–	74.42 ± 74.57	38.35 ± 36.79	0.101
AST (IU/l)	–	55.85 ± 43.86	40.21 ± 30.62	0.405
GGTP (IU/l)	–	35.25 ± 51.86	30.71 ± 74.70	0.243
WBC (G/l)		6.45 ± 2.10	8.64 ± 2.85	0.026*
Inflammatory activity (METAVIR)	–	1.85 ± 0.85	–	–
Fibrosis (METAVIR)	–	1.71 ± 0.94	–	–
Liver steatosis Y/N	9/17 (34.6/65.4)	–	–	–

$\bar {X}$ mean, *SD* standard deviation, *M* median, *BMI* body mass index, *Y* yes, *N* no, *G* 10^9^, *IU* international unit, *kg* kilogram

*Statistically significant p value

Liver biopsy specimens were taken in local anesthesia using Menghini technique (Braun). Histological evaluation was preformed according to METAVIR scoring system [12]. Clinical data of the patient were not revealed to the pathologist prior to histological assessment. AST to platelets ratio index (APRI) was used as indirect fibrosis marker. APRI was calculated according to formula: AST/UNLx100/PLT [13]. Values < 0.5 were excluding significant fibrosis, < 1.0—significant cirrhosis. While values exceeding 2.0 were suggestive for liver cirrhosis with good predictive value [13].

Cytometric testing was performed at the time of liver biopsy. NK cells were identified in patient PBMC as CD3^−^/CD56^+^, using monoclonal antibodies: anti-CD3 peridinin–chlorophyll–protein complex [PerCP, Becton Dickinson (BD) Biosciences, USA] and anti-CD56-allophycocyanin (APC Mouse anti-Human CD56, BD Biosciences, USA). The expression of NK cell surface antigens was evaluated using patient PBMC incubated with the following antibodies to inhibitory and activating receptors: anti CD158b (KIR2DL2/DL3)-phycoerythrin (PE) (human; clone DX27), anti-CD158e (KIR3DL1)-PE (human; clone DX9) and anti-CD314 (NKG2D)-PE (human; clone BAT221) (Miltenyi Biotec Inc). Mouse IgG1-PE, Mouse IgG2a-PE, Mouse IgG2a–APC (Miltenyi Biotec Inc.) were used as control kits. Background levels of staining was determined by isotype-matched controls. FACS lysing solution was used to lyse red cells and the lymphocytes were washed three times before further analysis on a FACS Canto flow cytometer (Becton Dickinson, USA). NK cell receptors were identified by flow cytometry and the results were presented as proportion of cells and mean fluorescence rate (MFI).

Children were treated with PEGIFN-α 2b (60 μg/m^2^ s.c 1×/week) and ribavirin (15 mg/kg p.o. daily). Duration of the treatment were response-related and varied from 12 to 72 weeks. Rapid virological response (RVR) was tested in the 4th week of the therapy and sustained virological response (SVR) was checked 6 months after completion of the therapy.

Control group consisted of 23 healthy children—12 boys and 11 girls, age 13.48 ± 5.14 years (*p* = 0.98) in whom only blood for hematological parameters (WBC, HGB, PLT), cytometry, and ALT and AST activity was taken (Table 1).

The study received the approval of the Bioethical Committee of the University of Medical Sciences in Poznań, Poland (no. 234/08 from September 4. 2008).

Continuous variables were expressed as mean, standard deviation, median and range. The Shapiro–Wilk test was used for normality assessment. Subsequently, values were compared with Student’s *t* test or Mann–Whitney tests where appropriate. Categorical variables were expressed as frequency and percentage. The Chi-square or Fisher exact test was used for the evaluation where appropriate. Correlations were assessed by Spearman-rank correlation test. Comparisons between multiple groups were performed using Kruskal–Wallis test. Factors with statistically significant differences between compared groups were included in further univariate analysis to evaluate their importance as predictors of response to the antiviral treatment. Logistic regression was performed by a model including factors found significant in univariate analysis. Values with *p* < 0.05 or with confidence interval (CI) not including 1.0 were defined as statistically significant.

## Results

**Fig. 1:**
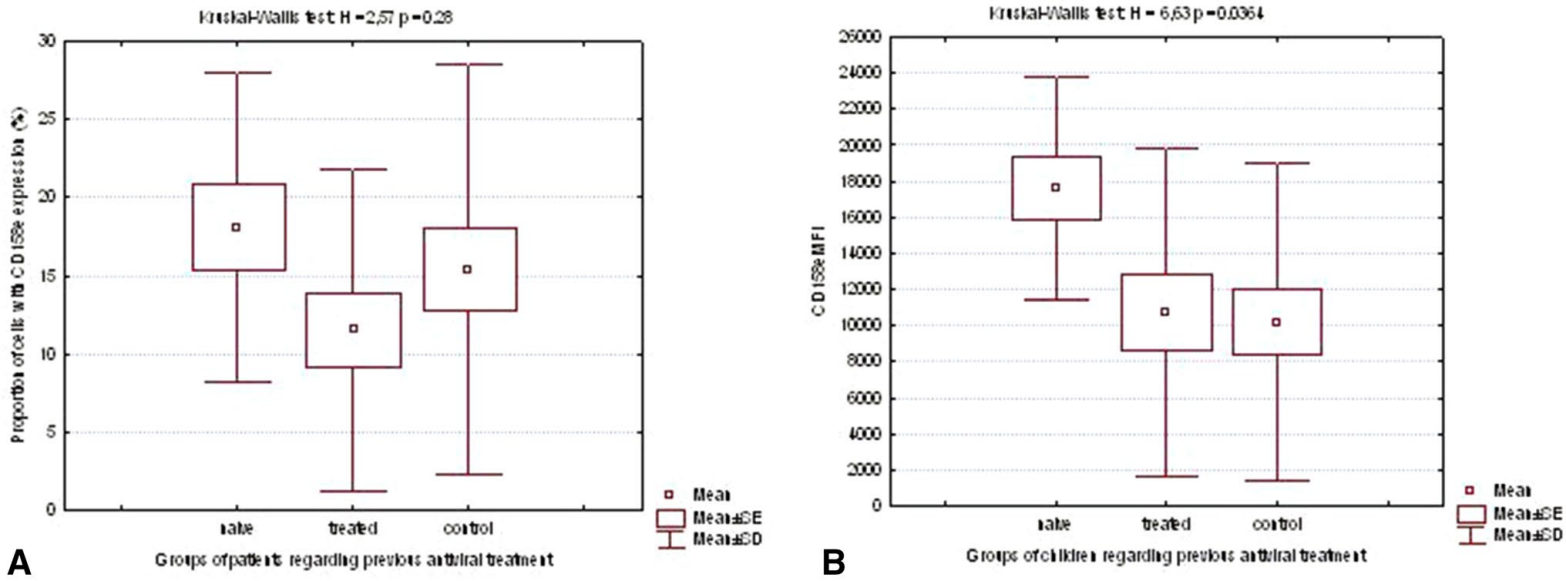
Proportion of cells with expression of CD158e (**a**) and the density of CD158e expression (**b**) in treatment-naïve and experienced patients with CHC

Half of the children from the study group underwent previous IFN-Rib treatment with negative outcome. In the group of treatment-naïve children, younger age (12.54 ± 4.24 vs 15.62 ± 0.87 years, *p* = 0.011), shorter duration of infection (8.23 ± 4.19 vs 13.38 ± 4.19 years, *p* = 0.001) was observed. Treatment-naïve children were more commonly infected on vertical mode and had no malignancy in the history (Table 2). This group of children developed lower viral load (1.79 × 10^5^±2.32 × 10^5^ vs 1.5 × 10^6^±1.58 × 10^6^ IU/ml, *p* = 0.007). Treatment-naïve children had higher proportion of NK cells expressing CD 158e (18.10 ± 9.92 vs 10.40 ± 8.45%, *p* = 0.044) as well as higher density of CD158b expression (logMFI = 3.54 ± 0.16 vs 3.40 ± 0.31 MFI, *p* = 0.045) (Table 2). Moreover, density of CD158e expression but not proportion of cells differed significantly in multiple comparison using Kruskal–Wallis test *H* = 6.63, *p* = 0.036; *H* = 2.57, *p* = 0.28, respectively (Fig. 1a, b).

**Table 2:** Baseline history data, laboratory and cytometric parameters in relation to previous antiviral treatment

Parameter	Treatment-naive *N* = 13 *X* ± SD	Previous antiviral treatment *N* = 13 *X* ± SD	*p*
Age (years)	12.54 ± 4.24	15.62 ± 0.87	0.011*
Gender: male/female	6/7	7/6	0.500
Age at diagnosis (years)	4.31 ± 4.46	2.23 ± 1.39	0.578
Duration of infection (years)	8.23 ± 4.19	13.38 ± 4.19	0.001*
Mode of transmission vertical/nosocomial	4/9	0/13	0.047*
Malignancy in the history Y/N	1/12	13/0	0.00001*
Weight (kg)	50.15 ± 18.30	62.57 ± 12.61	0.211
Height (m)	1.58 ± 0.19	1.70 ± 0.11	0.085
BMI (kg/m^2^)	19.31 ± 4.57	21.67 ± 3.02	0.166
ALT (IU/l)	91.31 ± 129.92	43.31 ± 30.81	0.336
AST (IU/l)	63.08 ± 68.46	35.92 ± 16.25	0.362
GGTP (IU/l)	21.00 ± 5.69	48.23 ± 79.79	0.222
HCV-RNA (IU/ml)	1.79 × 10^5^±2.32 × 10^5^	1.5 × 10^6^±1.58 × 10^6^	0.007*
Log (HCV-RNA)	4.32 ± 1.31	5.67 ± 0.92	0.006*
Genotype HCV 1a + 1b/3a	11/2	13/0	0.480
WBC (G/l)	7.20 ± 3.63	6.01 ± 1.27	0.273
NK (cell/μl)	398 ± 297	278 ± 162	0.124
NK (%)	14.79 ± 5.69	12.42 ± 5.43	0.229
CD158b (%)	33.86 ± 11.19	29.17 ± 12.67	0.169
Log (CD158b MFI)	3.54 ± 0.16	3.40 ± 0.31	0.045*
CD158e (%)	18.10 ± 9.92	10.40 ± 8.45	0.044*
Log (CD158e MFI)	4.22 ± 0.18	4.09 ± 0.28	0.350
CD158i (%)	17.49 ± 20.63	6.59 ± 16.22	0.223
Log (CD158i MFI)	4.04 ± 0.35	3.88 ± 0.07	0.857
KIR2D (%)	53.13 ± 13.34	43.83 ± 13.72	0.093
Log (KIR2D MFI)	4.36±	4.33±	0.238
NKG2D (%)	97.24 ± 3.41	95.10 ± 6.02	0.181
Log10 (NKG2D MFI)	3.64 ± 0.078	3.67 ± 0.088	0.311
Inflammatory activity (METAVIR)	1.42 ± 0.67	1.31 ± 0.48	0.852
Fibrosis (METAVIR)	1.58 ± 0.79	1.38 ± 0.77	0.650
Liver steatosis Y/N	5/8	4/9	0.500
APRI	1.17 ± 1.57	0.62 ± 0.41	0.237

*X* mean, *SD* standard deviation, *T* yes, *N* no

*Value of statistical significance (*p* < 0.05)

From the 26 children with CHC, 9 achieved RVR and 10 SVR. In 17 children, no RVR was detected and 16 children were considered nonresponders (NR) 6 month after completion of the therapy. The group of RVR children was younger (11.67 ± 3.74 vs 15.35 ± 2.42 years; *p* = 0.001) with shorter duration of infection (8.44 ± 4.19 vs 12.06 ± 4.23 years; *p* = 0.015) compared to children with no response. Children who reached RVR were more frequently infected on vertical mode, had no malignancy in the history and were treatment-naïve (Table 3). Moreover, RVR group presented higher expression of CD158b (logMFI = 3.58 ± 0.16 vs 3.45 ± 0.13; *p* = 0.038) and CD158e (logMFI = 4.33 ± 0.21 vs 4.03 ± 0.16; *p* = 0.039) compared to the group without RVR. No significant differences were observed between the groups in relation to biochemical parameters, viral load and liver histology. The results were presented in Table. 3.

**Table 3:** Baseline history data, laboratory parameters and liver histology in relation to RVR

Parameter	RVR *N* = 9 *X* ± SD	No RVR *N* = 17 *X* ± SD	*p*
Age (years)	11.67 ± 3.74	15.35 ± 2.42	0.001*
Gender: male/female	3/6	10/7	0.205
Age at diagnosis (years)	3.22 ± 3.70	3.29 ± 3.55	0.808
Duration of infection (years)	8.44 ± 4.19	12.06 ± 4.19	0.015*
Mode of transmission vertical/nosocomial	4/5	0/17	0.0084*
Malignancy in the history Y/N	1/8	13/4	0.0023*
Former antiviral treatment Y/N	0/9	13/4	0.0002*
Weight (kg)	50.50 ± 19.43	59.62 ± 14.69	0.190
Height (m)	1.56 ± 0.20	1.68 ± 0.13	0.07
BMI (kg/m^2^)	19.90 ± 4.96	20.80 ± 3.48	0.594
ALT (IU/l)	99.33 ± 155.94	50.35 ± 35.59	0.808
AST (IU/l)	70.78 ± 82.26	38.24 ± 15.51	0.935
GGTP (IU/l)	21.56 ± 6.77	41.53 ± 70.22	0.686
HCV-RNA (IU/ml)	2.32 × 10^5^±2.65 × 10^5^	1.18 × 10^6^±1.51 × 10^6^	0.145
Log (HCV-RNA)	4.52 ± 1.28	5.26 ± 1.28	0.145
Genotype HCV 1a + 1b/3a	7/2	17/0	0.111
WBC (G/l)	6.05 ± 1.43	6.90 ± 3.22	0.979
NK (cell/ul)	303 ± 221	333 ± 246	0.647
NK (%)	13.29 ± 5.80	13.78 ± 5.64	0.746
CD158b (%)	35.03 ± 10.19	29.65 ± 12.68	0.106
Log (CD158b MFI)	3.58 ± 0.16	3.45 ± 0.13	0.038*
CD158e (%)	17.84 ± 10.47	11.58 ± 9.40	0.194
Log (CD158e MFI)	4.33 ± 0.21	4.03 ± 0.16	0.039*
CD158i (%)	16.64 ± 21.02	9.61 ± 18.05	0.419
Log (CD158i MFI)	4.07 ± 0.36	3.17 ± 0.27	0.385
KIR2D (%)	54.42 ± 13.33	45.34 ± 13.81	0.125
Log (KIR2D MFI)	4.36 ± 0.07	4.34 ± 0.10	0.560
NKG2D (%)	97.30 ± 3.67	95.23 ± 5.79	0.104
Log (NKG2D MFI)	3.64 ± 0.08	3.67 ± 0.09	0.590
Inflammatory activity (METAVIR)	1.63 ± 0.74	1.24 ± 0.44	0.153
Fibrosis (METAVIR)	1.88 ± 0.83	1.29 ± 0.69	0.085
Liver steatosis Y/N	4/5	5/12	0.365
APRI	1.39 ± 1.89	0.63 ± 0.36	0.767

*X* mean, *SD* standard deviation, *T* yes, *N* no

*Value of statistical significance (*p* < 0.05)

**Fig. 2:**
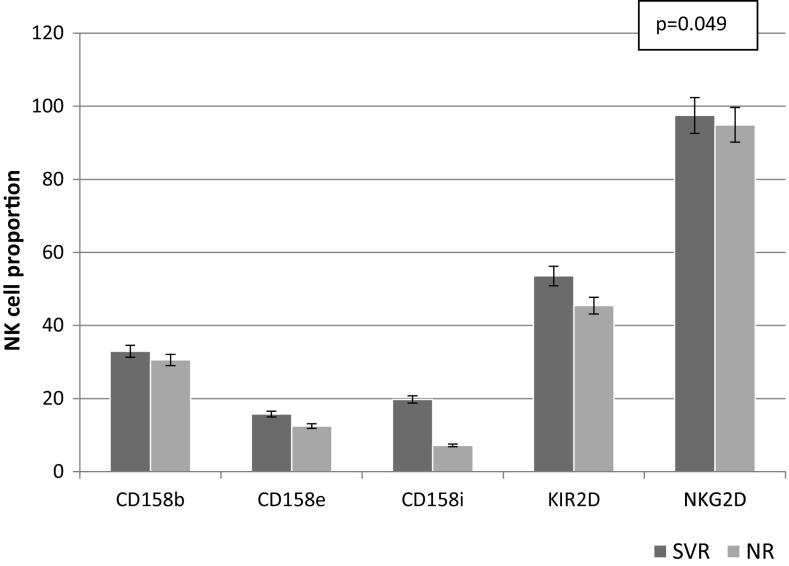
Baseline expression of activating and inhibitory receptors on the surface of NK cells in relation to SVR

In the SVR group, significantly younger age (12.40 ± 3.66 vs 15.13 ± 2.83 years; *p* = 0.003) and shorter duration of infection were observed (9.50 ± 4.48 vs 11.63 ± 3.88 years; *p* = 0.015). Children vertically infected and not treated previously were also more common in this group of patients (Table 4). Thus, no difference was noticed between the groups in relation to the history of malignancy. No differences were observed between the groups in relation to aminotransferase activity, viral load and HCV genotype. Furthermore, the density of CD15b (logMFI = 3.68 ± 0.22 vs 3.36 ± 0.16; *p* = 0.036) and CD158e expression was significantly higher (logMFI = 4.37 ± 0.14 vs 4.12 ± 0.21; *p* = 0.046) in children with SVR. The proportion of NK cells with the expression of NKG2D was also significantly higher (97.50 ± 3.46 vs 94.92 ± 5.93; *p* = 0.049) in this group of children. Whereas, the density of NKG2D expression measured as MFI was lower in patients with SVR (Fig. 2). The difference was, however, not statistically significant. The group with SVR compared to children with no response to treatment did not differ significantly regarding biochemical parameters, viral load and liver histology (Table 4).

**Table 4:** Baseline history data, laboratory parameters and liver histology in relation to SVR

Parameter	SVR *N* = 10 *X* ± SD	NR *N* = 16 *X* ± SD	*p*
Age (years)	12.40 ± 3.66	15.13 ± 2.83	0.003*
Gender: male/female	5/5	8/8	0.656
Age at diagnosis (years)	2.90 ± 3.63	3.50 ± 3.56	0.407
Duration of infection (years)	9.50 ± 4.48	11.63 ± 3.88	0.015*
Mode of transmission vertical/nosocomial	4/6	0/16	0.014*
Malignancy in the history Y/N	3/7	11/5	0.063
Former antiviral treatment Y/N	2/8	11/5	0.021*
Weight (kg)	59.65 ± 21.00	54.47 ± 13.73	0.692
Height (m)	1.63 ± 0.19	1.64 ± 0.15	0.874
BMI (kg/m^2^)	21.63 ± 5.07	19.77 ± 3.09	0.475
ALT (IU/l)	98.90 ± 146.50	47.56 ± 36.11	0.189
AST (IU/l)	68.10 ± 78.05	37.88 ± 15.85	0.142
GGTP (IU/l)	21.10 ± 6.94	43.06 ± 72.20	0.351
HCV-RNA (IU/ml)	7.65 × 10^5^±1.18 × 10^6^	9.57 × 10^5^±1.44 × 10^6^	0.718
Log (HCV-RNA)	4.90 ± 1.39	5.10 ± 1.28	0.718
Genotype HCV 1a + 1b/3a	8/2	16/0	0.138
WBC (G/l)	6.15 ± 1.31	6.89 ± 3.35	0.752
NK (cell/μl)	306 ± 214	333 ± 251	0.776
NK (%)	12.50 ± 5.44	14.30 ± 5.73	0.279
CD158b (%)	32.96 ± 11.13	30.61 ± 12.71	0.414
Log (CD158b MFI)	3.68 ± 0.22	3.36 ± 0.16	0.036*
CD158e (%)	15.77 ± 9.86	12.49 ± 10.26	0.510
Log (CD158e MFI)	4.37 ± 0.14	4.12 ± 0.21	0.046*
CD158i (%)	19.77 ± 22.15	7.21 ± 15.61	0.162
Log (CD158i MFI)	3.83 ± 0.01	3.97 ± 0.32	0.800
KIR2D (%)	53.55 ± 14.37	45.44 ± 13.45	0.188
Log (KIR2D MFI)	4.32 ± 0.06	4.35 ± 0.10	0.640
NKG2D (%)	97.50 ± 3.46	94.92 ± 5.93	0.049*
Log (NKG2D MFI)	3.60 ± 0.11	3.69 ± 0.09	0.160
Inflammatory activity (METAVIR)	1.60 ± 0.70	1.20 ± 0.41	0.102
Fibrosis (METAVIR)	1.80 ± 0.70	1.27 ± 0.70	0.083
Liver steatosis Y/N	5/5	4/12	0.189
APRI	1.33 ± 1.78	0.62 ± 0.38	0.916

*X* mean, *SD* standard deviation, *T* yes, *N* no

*Value of statistical significance (*p* < 0.05)

Univariate analysis confirmed significance of the age and duration of infection as negative predictors of RVR. Moreover, the expression of CD 158b and CD158e was found a positive predictor of RVR. All these parameters were found significant factors predicting RVR in multivariate analysis (Table 5).

**Table 5:** Univariate and multivarite analysis as predictors of RVR

Factor	Univariate analysis		Multivariate analysis	
	OR (95% CI)	*p*	OR (95% CI)	*p*
Age (years)	0.61 (0.38–0.99)	0.037*	0.62 (0.59–0.65)	0.0001*
Duration of infection (years)	0.79 (0.63–0.99)	0.045*	0.91 (0.88–0.95)	0.000001*
Vertical mode of infection	0.99 (0.18–54.26)	0.995	–	–
Malignancy in the history	0.98 (0.18–5.37)	0.986	–	–
Former antiviral treatment	0.98 (0.17–5.76)	0.987	–	–
Log (CD158b MFI)	11.42 (538.17–2280.58)	0.0047*	14.41(51.13–406.57)	0.000001*
Log (CD158e MFI)	23.44 (13.97–39.32)	0.00001*	6.96(3.47–13.96)	0.00000004*

*OR* odds ratio, *CI* confidence interval, *MFI* mean fluorescence intensity

*Statistically significant *p* value (*p* < 0.05)

Significance of the age of patients and the expression of CD158b and CD158e as predictors of SVR were confirmed in the univariate analysis. While the expression of CD158b and CD158e were found positive predictors in further multivariate analysis (Table 6).

**Table 6:** Univariate and multivarite analysis as predictors of SVR

Factor	Univariate analysis		Multivariate analysis	
	OR (95% CI)	*p*	OR (95% CI)	*p*
Age (years)	0.82 (0.79–0.84)	0.0003*	0.94 (0.87–1.02)	0.14
Duration of infection (years)	0.88 (0.71–1.08)	0.211		–
Vertical mode of infection	0.99 (0.12–8.11)	0.995	–	–
Malignancy in the history	0.99 (0.12–8.13)	0.993	–	–
Former antiviral treatment	0.99 (0.13–7.64)	0.993	–	–
Log (CD158e MFI)	4.76 (2.75–8.27)	0.0000001*	13.23(5.39–32.50)	0.0000002*
Proportion of cells with NKG2D (%)	1.07 (1.04–1.11)	0.000001*	1.11(1.08—1.14)	0.00003*

*OR* odds ratio, *CI* confidence interval, *MFI* mean fluorescence intensity

*Statistically significant p value (*p* < 0.05)

## Discussion

In the era of DAA, treatment of children with CHC is still based on PEG + IFN–RBV. Although children were proven to be the same candidates for antiviral treatment as adult patients having much less comorbidities complicating the therapy, none of the newly registered oral drugs were tested on pediatric population [14]. Combined PEG–IFN/RBV therapy gives 50–90% chance of HCV clearance [14, 15]. Nevertheless, still substantial proportion of patients fails to achieve SVR. In current study, SVR rate was 38.5% which is significantly lower compared to available results [14, 15]. Study group consisted of children who were already nonresponders to previous therapy attempts (50%). Although SVR group consisted more frequently of treatment-naïve children. Previous antiviral therapy was not found to be a negative predictor of response to current PEG–IFN/RBV treatment. It has to be stressed that in our study the age of patients was found to be an imperative predictor of the RVR and SVR negatively related to the treatment outcome. Therefore, the best therapy results are expected in young patients.

Numerous indicators of immune response that may be useful as predictors of the effect of PEG–IFN/RBV therapy were described [8, 9]. NK cell phenotype was found to be associated with treatment outcome in patients with CHC. Oliviero et al. described different NK-cell phenotypic and functional features suggesting a potential role of NK cells in the response to PEG–IFN/RBV treatment. In their study, important phenotypic differences between RVR and NR were already present prior to the treatment commencement [8].

In the current study, pretreatment evaluation of NK cell receptors in children with CHC revealed inhibitory phenotype. Proportion of cells with CD158e expression as well as the density of expression of this receptor was found significantly higher in the group of treatment-naïve children. Moreover, the CD158e expression was higher as well as higher proportion of cells with the expression of activating receptor NKG2D in SVR group. The pretreatment expression of CD158e was a positive predicting factor of SVR in both univariate and multivariate analysis. Proportion of cells with the expression of CD158e receptor correlated to the liver injury expressed as ALT activity and APRI index, which is consistent with our previous findings [16]. Umemura et al. in their study regarding adult Japanese patients with CHC also found the expression of this receptor and its ligand HLA-Bw4 an independent predictor of SVR [17]. Our study consisted however of a group of patients of different age and ethnicity.

Our findings are not consistent with Golden-Mason et al. [4], who found that increased expression of inhibitory receptors CD158b, CD158e and NKG2A were negatively related to SVR during interferon-based therapy. However, raised proportion of cells with the expression of NKR44p, TRAIL CD161 was positively correlated to this type of treatment response [18]. On the other hand, relation between the expression of inhibitory receptor CD158b and its ligand MHC-C1 and spontaneous elimination of HCV as well as the response to antiviral treatment [19] were noticed by Knapp et al. Vidal-Castiniera described increased frequency of genotype of this receptor in adult patients that achieved SVR [20]. Data concerning the expression of KIR receptors in relation to antiviral treatment in children are limited.

Sene et al. describe decreased expression of the NKG2D activating receptor on NK cells as HCV strategy to evade natural immune response mechanisms, since NK cell-mediated cytotoxic capacity and interferon-γ production are impaired as a consequence [21]. Therefore, higher NKG2D expression in SVR patients may favor good PEG–IFN/RBV treatment outcome as was observed in our study. Regulation of NKG2D expression may be multifactorial and influenced by the host and the virus [21]. In our study, HCV genotype did not influence on the expression NK cell receptors.

Furthermore, Golden-Mason et al. noticed, that, activated phenotype of NK cells was not reversed by successful IFN-alpha-based therapy and was expressed as greater lymphokine-associated killing activity, viral control and degranulation [22] Moreover, antiviral treatment with IFN-alpha ribavirin was found to restore the activity of NK cells increasing the frequency of CD56 (bright) NK cells in CHC patients that achieved SVR [23]. PEG–IFN was found to activate NK cells inducing their cytotoxic function, which however correlated to the treatment response [24]. The restoration of NK-cell activity was assumed to be a result of HCV viral load reduction by PEG–IFN/RBV treatment [23]. In current study, NK cell receptors were analyzed only at baseline to evaluate initial predictors of successful outcome. Therefore, no alterations after the treatment could be observed. Further analysis would be required to observe alterations in NK cell receptors expression in the course of the therapy. However, we did not observe any relation between HCV-RNA and expression of NK cell receptors in our study.

Interferon-free regimens were also described to influence intrahepatic immune activation. HCV clearance achieved by interferon-free regimens was found to be associated with loss of intrahepatic immune activation by IFN-alpha. Decreased levels of CXCL10 and CXCL11 along with a normalization of NK cell phenotype and function were observed until the end of the therapy [25]. Other authors describe that during the treatment with novel DAA, decreased production of NK cell-stimulating cytokines was noticed which caused normalization of altered NK cell phenotype in the course of CHC [26]. Nevertheless, since DAA regimens are not available for children, these observations cannot be verified in this group of patients.

## Conclusions

Age of patients is negatively related to RVR and SVR; therefore, young patients should be enrolled into treatment strategies. NK cell phenotype with higher expression density of CD 158b and CD158e receptor was a positive predictor of SVR.
